# Video-EEG monitoring as a valuable tool for antiseizure medication withdrawal in patients with epilepsy: implications for clinical practice and public health policies

**DOI:** 10.1186/s42466-023-00278-0

**Published:** 2023-09-28

**Authors:** Obed Okwoli Apochi

**Affiliations:** Neuropsychology, School of Behavioral Forensics, National Forensic Sciences University, Gandhinagar, Gujarat India

**Keywords:** Video-EEG, Antiseizure, Withdrawal, Epilepsy, Public Health

## Abstract

This letter to the editor discusses “the use of video-EEG monitoring to guide antiseizure medication (ASM) withdrawal in patients with epilepsy” [1]. The author highlights the potential benefits of this approach, including reduced risk of seizure recurrence and improved patient outcomes. The author also notes the need for further research to refine the criteria for identifying patients who are good candidates for ASM withdrawal and to evaluate the effectiveness of this approach in different patient populations and settings. Finally, the author discusses the implications of these findings for public health policies related to epilepsy management.

Dear Editor,

I read with great interest the recent article by Dhaenens-Meyer et al. [1] on “The Use of Video-EEG Monitoring to Guide Antiseizure Medication (ASM) Withdrawal in Patients with Epilepsy.“ As a Neuropsychology student with ardent interest in epilepsy, I believe that these findings hold profound implications for optimizing epilepsy management.

One of the primary challenges in epilepsy care revolves around the safe and appropriate withdrawal of ASM. While ASM can effectively control seizures, it may entail significant side effects and financial burden for patients [1]. Additionally, long-term use of ASM can lead to drug resistance, rendering it unnecessary for certain individuals. Therefore, the identification of suitable candidates for ASM withdrawal and a safe withdrawal process are vital considerations [4].

The study by Dhaenens-Meyer et al. [1] suggests that video-EEG monitoring can be a valuable tool in tackling this challenge. By continuously monitoring seizure activity during the tapering process, healthcare providers can make more informed decisions about the optimal timing and pace for ASM withdrawal. The study revealed that EMU-guided ASM withdrawal was successful in 90.9% of cases, and the sensitivity of the LPM for a 2-year 50% relapse risk threshold was 75%, underscoring the model’s potential as a risk assessment tool for patients who have achieved long-term seizure freedom [1].

Nonetheless, as the authors astutely acknowledge, further research is warranted to fully comprehend the effectiveness of this approach and to refine patient selection criteria for ASM withdrawal. Future studies should explore the utility of video-EEG monitoring in diverse patient populations and settings. For instance, investigations could delve into the model’s applicability in pediatric epilepsy or drug-resistant epilepsy cases, providing deeper insights into its broader suitability.

Comparative analyses between video-EEG monitoring and other ASM withdrawal methods could also shed light on the model’s unique advantages. In this regard, Lamberink et al. [3] presented a prediction model for estimating the risk of seizure recurrence after ASM withdrawal. Building upon their work could help refine the criteria for identifying optimal candidates for ASM withdrawal and may assist in devising personalized withdrawal strategies [3].

Moreover, the implications of the study’s findings stretch beyond clinical practice and extend to public health policies related to epilepsy management. Existing policies that prioritize ASM access might necessitate reevaluation in light of the possibility of ASM withdrawal. Policymakers should contemplate how to best support individuals with epilepsy who opt for ASM withdrawal, including the provision of resources to manage withdrawal symptoms and continuous monitoring of seizure activity during the withdrawal process [2].

In conclusion, the study by Dhaenens-Meyer and colleagues highlights the potential benefits of incorporating video-EEG monitoring into epilepsy management. By offering a more objective and data-driven approach to ASM withdrawal, video-EEG monitoring could substantially mitigate the risk of seizure recurrence and improve overall patient outcomes. I am optimistic that ongoing research will further explore and enhance this approach, and I strongly encourage healthcare providers to consider implementing video-EEG monitoring when appropriate.

Sincerely,

Apochi Obed Okwoli.

## Data Availability

Not Applicable.
